# Determinants of performance of supplemental immunization activities for polio eradication in Uttar Pradesh, India: social mobilization activities of the Social mobilization Network (SM Net) and Core Group Polio Project (CGPP)

**DOI:** 10.1186/1471-2334-13-17

**Published:** 2013-01-17

**Authors:** William M Weiss, Md Hafizur Rahman, Roma Solomon, Dora Ward

**Affiliations:** 1 Department of International Health, Johns Hopkins Bloomberg School of Public Health, 615 North Wolfe Street, Suite E8132, Baltimore, MD, 21205, USA; 2 CORE Group Polio Project – India, 45/201 Heritage City, MG Road, Gurgaon, India; 3 CORE Group Polio Project, 151 Ellis Street, NE, Atlanta, GA, 30303, USA

## Abstract

**Background:**

The primary strategy to interrupt transmission of wild poliovirus in India is to improve supplemental immunization activities (SIAs) and routine immunization coverage in priority districts. The CORE Group, part of the Social Mobilization Network (SM Net), has been successful in improving SIA coverage in high-risk areas of Uttar Pradesh (UP). The SM Net works through community level mobilisers (from the CORE Group and UNICEF) and covers more than 2 million children under the age of five. In this paper, we examine the reasons the CORE Group had been successful through exploration of which social mobilization activities of the CORE Group predicted better performance of SIAs.

**Methods:**

We carried out a secondary data analysis of routine monitoring information collected by the CORE Group and the Government of India for SIAs. These data included information about vaccination outcomes of SIAs in CORE Group areas and non-CORE Group areas within the districts where the CORE Group operates, along with information about the number of various social mobilization activities carried out for each SIA. We employed Generalized Linear Latent and Mixed Model (GLLAMM) statistical analysis methods to identify which social mobilization activities predicted SIA performance, and to account for the intra-class correlation (ICC) between multiple observations within the same geographic areas over time.

**Results:**

The number of mosque announcements carried out was the most consistent determinant of improved SIA performance across various performance measures. The number of *Bullawa Tollies* carried out also appeared to be an important determinant of improved SIA performance. The number of times other social mobilization activities were carried out did not appear to determine better SIA performance.

**Conclusions:**

Social mobilization activities can improve the performance of mass vaccination campaigns. In the CORE Group areas, the number of mosque announcements and *Bullawa Tollies* carried out were important determinants of desired SIA outcomes. The CORE Group and SM Net should conduct sufficient numbers of these activities in support of each SIA. It is likely, however, that the quality of social mobilization activities (not studied here) is as or more important than the quantity of activities; quality measures of social mobilization activities should be investigated in the future as to how they determine vaccination performance.

## Background

In 1988, the estimated number of wild poliovirus in 1988 was 350,000 [1]. However, by the end of 2010, the total number of wild polio cases fell to 1288 [2]. As of 7 June 2011, the total number of 2011 wild polio cases worldwide was 195 and there was only one (1) reported case of wild poliovirus in India compared to 43 and 741 in all of 2010 and 2009, respectively [2-4]. Although India interrupted transmission of wild poliovirus in 2012, India remains at risk of an importation of wild poliovirus from neighboring Pakistan, similar to the recent importation in China—a country polio free since 1994 [5]. 

The majority of wild polio cases in India have been in the states of Uttar Pradesh and Bihar [4]. The strategy to interrupt transmission of wild poliovirus in India is to conduct frequent supplemental immunization activities (SIAs or mass campaigns) in high-risk districts and blocks. The high frequency of campaigns is designed to overcome “high immunity thresholds,” meaning that an extremely high percent of the population needs to have immunity in order to interrupt transmission [6]. During an SIA, oral polio vaccine (OPV) is given to all children in the target group of 0–5 years as a part of the polio eradication program. The yearly frequency of SIAs in India may vary from 4–12 and the scope can range from a district to an entire state up to the entire country. Grassroots social mobilization efforts, including those of the CORE Group and SM Net, have been effective in reaching underserved populations during SIAs and combating rumors against polio vaccination in India [7-10].

The CORE Group is a US-based organization made up of health professionals, working for a variety of non-governmental organizations, to collaborate on international health and development programs [11]. In India, the CORE Group Polio Project (CGPP), with funding from the US Agency for International Development (USAID), works in ten districts of the state of Uttar Pradesh (UP) through a consortium of the following PVOs: Adventist Development & Relief Agency (ADRA) India, PCI and Catholic Relief Services (CRS), as well as their local NGO partners.^a^ The CGPP in India has an extensive network of 1,325 Community Mobilization Coordinators (CMCs) who conduct social mobilization activities for behavior change related to polio vaccination. These CMCs are a part of the Social Mobilization Network (SM Net) in India that includes CGPP, UNICEF, Rotary, and the Indian Government’s and WHO’s National Polio Surveillance Project (NPSP). The SM Net was formed in UP in 2003 to support polio eradication efforts there by: identifying high-risk areas and working with underserved communities in planning, implementing and monitoring social mobilization and other immunization activities in those high-risk areas. The three-tier network of community mobilizers (community level, block level, and district level) does the main work of the SM Net.

The Community Mobilization Coordinator (CMC) interacts with families and community members at the village level. As the backbone of the SM Net, s/he is assigned responsibility for mobilizing about 500 households in either a rural or an urban area.S/he keeps records of the immunization status of all children less than five years of age in those households. CMC areas are groups of communities within a block where the SM Net deploys CMCs. The SM Net selects these communities for additional social mobilization efforts based on past communication-related and operational challenges for immunizing children. Most of the CMCs are deployed in areas designated as High Risk Areas (HRAs). Jointly with key partners (Unicef, MOH and CGPP), NPSP defines the criteria for HRAs; these criteria are reviewed periodically and modified. The most recent criteria for HRAs take into account the following information: the number of wild polio virus (P1) cases during low transmission seasons since 2003; the presence of High Risk Groups (Slum dwellers/Nomads); the number of cases last two years with polio-like symptoms; if 40% or more of the population is Muslim; and, the percent of households that have unvaccinated children (X houses). Once a community is identified as an HRA, the SM Net arranges for CMCs to work there. A CMC has to be 18 years or more, preferably female and from the same community. The partnership periodically revises the areas designated as an HRA. All CMCs are paid a monthly honorarium of Rs. 1600 (about $30-35).

During SIA (Supplementary Immunization Activity) rounds, CMCs do the following: assist vaccinators in setting up vaccination booths; organize groups of child mobilizers (*Bullawa tollies*); help arrange for mosque and/or temple announcements, rallies, interpersonal communication meetings, and meetings with influential people. CMCs also accompany vaccinator teams to homes with children under five years of age, work to convince families with an unvaccinated child (called an ‘X’ household) to allow their child to be vaccinated (called converting an ‘X’ household to ‘P’, with ‘P’ denoting a house where all eligible children are vaccinated against polio), and accompany persons of influence (influencers) during home visits. Conversion of ‘X’ households to ‘P’ is measured during each SIA round. The Block Mobilization Coordinator (BMC) oversees social mobilization activities during (and in between) SIA rounds through supervision and mentoring of the CMCs working in the block. A description of key social mobilization activities of the CGPP and the SM Net is provided below.

### *Bullawa tollies*

One of the most interesting activities that CMCs conduct harnesses the potential of schoolchildren. S/he conducts ‘polio classes’ at schools in her/his area promoting hand washing and cleanliness in a fun way. In these classes, s/he also uses various methods to get the children interested in becoming a part of the polio campaign---from poetry and painting competitions on the polio theme to rallies. A few children are then selected to come together as ‘*Bullawa Tollies*’ (Literal translation = Calling gangs). These children (ages between 5 and 12 years) visit homes throughout the neighborhood during booth activities, not only persuading mothers to have their infants taken to booths for immunization but also to bring the babies themselves and be rewarded with small token gifts.

### Mosque and temple announcements

These announcements remind families about the SIA date and increase program credibility when delivered to a religious congregation. Most places of worship now have a PA system and this amplifies the reach of the message. The CMC contacts the mosque/temple priest and asks him to deliver the messages and thus participate in the program.

### Rallies

CMCs approach schools about recruiting children to participate in rallies held the day before the SIA. During the rallies, children spread the word about the SIA. The go around their village carrying placards that show date and making verbal announcements.

### Influencer meetings

The purpose of “Influencers Meetings” is for CMCs to obtain the cooperation of influential persons such as, community leaders, religious leaders, practitioners of alternate medicine including “quacks” or illegitimate practioners, ration dealers, shopkeepers, etc. Through demonstrating their support for polio vaccination efforts, influencers can help gain community support for the CMCs and act as a credible communication channel for the community. Ideally, the CMCs use the meetings to convince influencers to visit homes with the CMCs during SIA rounds. The influencers assist the CMCs to allay fears of families who are reluctant to vaccinate their children for various reasons (e.g., illness of child, fear that child is too young for vaccination, fear of sickness resulting from vaccination, etc.). CMCs also use these meetings to convince religious leaders to make encouraging SIA announcements from mosques and temples prior to the campaigns. These persons’ participation is voluntary.

### Interpersonal communication (IPC) meetings

The individual-focused activity performed by CMCs in between SIA rounds is Interpersonal Communication (IPC) Meetings with mothers and caregivers, especially with those who express resistance to vaccination. The purpose of IPC Meetings is to address misconceptions, rumors and fear through face to face dialogue. During IPC Meetings, the CMC shares information about polio: how the virus is transmitted, and how transmission can be prevented. S/he promotes routine immunization as well as immunization during each mass immunization campaign.

In this paper, we explore the reasons vaccination outcomes were found to be better in CMC areas than in non-CMC areas, as described in Weiss et al. (2011) [7]. We explore which social mobilization activities predict better or worse performance in CMC areas as compared to Non-CMC areas. The purpose is to identify which social mobilization should be continued and which ones should not. Much effort and many resources are being used to carry out social mobilization activities in support of polio eradication. Information that can help program managers rationalize which should be continued, among many social mobilization activities, will help improve the cost-effectiveness of polio eradication efforts.

## Methods

### Study design

This study is a secondary analysis of data originally collected for the purpose of program management. The original data include information about each SIA round across the CMC areas of all blocks that the CGPP works in (e.g., there was no sampling of sites within the CGPP program area). Given the type of data available for this paper, we chose to model the number of social mobilization activities (as covariates) against several vaccination outcomes---to identify which activities appear predictive of improved outcomes.

### Description of data

As described in more detail in Weiss et al. (2011), the original data represent programmatic monitoring data collected by CMCs during the course of their ongoing work [7]. Of interest to this analysis, the CGPP tracks two important indicators of social mobilization performance during SIAs: (1) Booth Coverage; and, (2) Percent of “X” households converted to “P”. Booth Coverage is the proportion of eligible children that were vaccinated at vaccination booths. The denominator is the total number of children vaccinated during the previous SIA (at booths and during house-to-house visits by vaccinators), and therefore varies between SIAs. CMC activity should increase Booth Coverage by motivating families to take their children to vaccination booths located in their own communities. The definition of an “X” household is discussed above. CMC activity should also increase the Percent of “X” households converted to “P.” The reasons for a household to have unvaccinated children (to be an “X” household) could include overt refusals by caregivers, sick children, children not present during vaccination, or locked houses. The CMC works to convince resistant parents and parents of sick children to get their children vaccinated.

The CMCs and BMCs plan their social mobilization activities prior to each SIA and document them in their own ‘planning registers’. These then become a part of the District Communication Plan shared at the District Task Force Meeting held prior to each SIA. The CGPP maintains a central database of these data that have been compiled and cleaned for this secondary data analysis. Each record/row in the database contains information about vaccination performance during a single SIA (organized by month) in one block. Each row of the database represents the aggregate performance of one SIA for either the CMC area or the Non-CMA area of a block. CMC areas cover roughly one third of the entire block while non-CMCs areas account for the remaining two-thirds. Record/rows in the database include the following information: district in which the SIA was conducted; block in which the SIA was conducted; whether the record represents a CMC or non-CMC area; the month and year of the SIA; the estimated number of children under age of five years living in the areas that the record represents; the performance indicators described above; and, the number of the various social mobilization activities. At the time of this writing, CGPP India was working in ten districts. The data were consolidated for these districts for the period January 2008 through September 2009; data from earlier SIAs are in the database but are not considered to be of sufficient reliability or quality to include in the analysis.

The districts and blocks included in this analysis (for the period 2008–2009) are listed in Table 1. Table 1 also shows the number of vaccination campaigns each block contributes to the analysis. The number of campaigns per block ranges from seven to 17. The mean number of children under five living in the CMC area of a block across the project and by district is shown in Table 2. Also included in Table 2 is the median number of social mobilization activities carried out in the CMC area of block across Supplemental Immunization Activities (SIAs) during the 2008–2009 period of this study.

**Table 1:** Blocks included in the analysis by district and number of polio vaccination campaigns, 2008-2009

**District**	**Block**	**Number of campaigns**	**District**	**Block**	**Number of campaigns**
Baghpat	Baghpat	15	Muzafarnagar	Baghra	17
				Budhana	17
	Baraut	15		Charthawal	17
	Binauli	15		Jansath	17
	Chaproli	15		Khatauli	17
	Khekhra	15		Shamli	17
	Pilana	15		Un	17
Bareilly	Baheri	17	Rampur	Bilaspur	17
	Bhojipura	17		Chamrua	17
	Dalelnagar	17		Swar	17
	Meerganj	17		Tanda	17
	Nawabganj	17			
Mau	Ghosi	15	Saharanpur	City	15
	Kopaganj	15		Nakur	15
	Pardaha	15		Sarsawan	15
	Ranipur	8		Sunehty	15
	Ratanpura	7			
Meerut	Hastinapur	17	Shahjahanpur	Bhawalkherha	17
	Kharkhauda	17		Jaitipur	17
	P. Garh	17		Kalan	17
	Rohta	17		Mirzapur	17
	Sardhana	17		Sindhauli	17
Moradabad	Bhojpur	17	Sitapur	Biswan	16
	M. Pandey	17		Machrehata	16
	Manota	17		Persendi	16
	Naroli	17		Pisawan	16
	Panwasa	17		Reusa	15
	Sambhal R	17		Sanda	15
	Sambhal U	17			
	Zone - 3	17			
	Zone - 4	17			
	Zone - 5	17			

**Table 2:** Mean number of children under five and median number of social mobilization activities in CMC areas of blocks across supplemental immunization activities (SIAs) by District, 2008-2009

			**Social Mobilization Activities in CMC Areas of Blocks**					
**District**		**Children under 5 yrs**	**Mosque Announcements**	**Temple Announcements**	**Bullawa Tollies**	**Influencer Meetings**	**Rallies**	**IPC Meetings**
		**(Mean)**	**(Median)**	**(Median)**	**(Median)**	**(Median)**	**(Median)**	**(Median)**
All Districts		11781	31	2	33	18	20	642.5
By District:								
1	Baghpat	11775	30	3	36	24	21	437
2	Bareilly	9212	16	1	20	10	19	400
3	Mau	9986	13	6	41	18	20	537
4	Meerut	9096	42	4	34	11	17	557
5	Moradabad	17051	57	3	39	24	25	1572
6	Muzafarnagar	13475	52	2	30	12	19	704
7	Rampur	9864	38	2	23	5	14	588
8	Saharanpur	9507	33	2	36	18	19	1170
9	Shahjahanpur	11455	8	4	20	18	19	648
10	Sitapur	9141	3	0	59	20	20	581

### Statistical analysis

We employed Generalized Linear Latent and Mixed Model (GLLAMM) to account for the intra-class correlation (ICC) between observations within the blocks as it may statistically contribute to an underestimation of standard error, which increases the likelihood of rejecting the null hypothesis committing a Type 1 error [12]. GLLAMM provides statistically efficient estimates of regression coefficients by correcting the standard errors, and allows the exploration of variation at different levels of hierarchy [13]. We conducted multivariate analysis to measure the association between difference in performance between the CMC and non-CMC areas of a block (outcome variable) and the numbers of social mobilization activities carried out in the CMC area of a block (independent variables of interest). The social mobilization activities were only carried out in the CMC areas. We multicollinearity checks among the social mobilization activities (number of Mosque Announcements, number of Rallies and number of *Bullawa Tollies*) and highly correlated variables (>0.80) were not included in the multivariate models.

For the missing data, in case of missing >0.05, imputation was done by putting the same data from the adjacent cases after sorting. For example, if social mobilization data is missing for a block for a particular month, data from the adjacent case (before or after) in the database was imputed after sorting by block and month.

We calculated the difference in performance between the CMC and non-CMC areas of a block. We did this for the indicators being assessed (booth coverage, conversion of X house to P) for each block and for each SIA. We then used these differences in performance as the outcome variables for this analysis. The numbers of social mobilization activities carried out in the CMC area of a block were the covariates in this analysis. We created an indicator variable from the numbers of social mobilization activities using a quartile distribution to provide four evenly distributed indicator values (1 = <25th percentile; 2 = 25th-50th percentile; 3 = 50th-75th percentile; and, 4= > 75th percentile). In Table 3, the distribution of the quartile values for the number of social mobilization activities is provided. The quartile values (1–4) were used as covariates in the analysis.

**Table 3:** Quartile distribution of the number of social mobilization activities in CMC areas of blocks and indicator value for covariates used in analysis

		**Number of Social Mobilization Activities**					
**Quartile | Percentile**		**Mosque Announcements**	**Temple Announcements**	**Bullawa Tollies**	**Influencer Meetings**	**Rallies**	**IPC Meetings**
1	< 25th	< 13	1	< 22	< 10	< 16	< 468
2	25 – 50th	13 – 31	2	22 – 33	10 – 18	16 – 20	468 – 642
3	50 – 75th	31 – 49	2 – 5	33 – 44	18 – 23	20 – 23	642 – 1060
4	> 75th	> 49	>5	> 44	> 23	> 23	> 1060

We assumed that performance at the block level might vary significantly between districts and by the number of children the program was trying to reach in a block. To test these possibilities, we included the program district (as an indicator variable), the number of children less than five years of age in the CMC areas of a block, and the number of social mobilization activities in the CMC areas of a block (as quartile indicator variables) in a multivariate statistical analysis using STATA statistical software [14].

## Results

### Determinants of the difference in booth coverage between CMC and Non-CMC areas

Determinants of the difference in booth coverage between CMC and Non-CMC areas are shown in Table 4. Variables originally entered into the GLLAMM analysis include the following: number of estimated children less than five years of age living in the block during the SIA; district where the SIA took place; number of IPC meetings carried out in preparation for the SIA; number of influencer meetings carried out in preparation for the SIA, number of rallies, number of mosque announcements, number of temple announcements, and number of *Bullawa Tollies*. The number of mosque announcements, *Bullawa Tollies*, and rallies were significant social mobilization determinants of the difference in booth coverage between CMC and Non-CMC areas. Unexpectedly, the relationship between the number of rallies and the difference in booth coverage was negative. The number of children under five years of age and the district also were significant determinants of the difference in booth coverage between CMC and Non-CMC areas.

**Table 4:** Determinants of the difference in booth coverage between CMC and non-CMC areas of a block*

**Variable**			**Coefficient**	**Std. Err.**	**z**	**p value**	**[95% Confidence Interval]**	
(Constant)			29.51482	3.560009	8.29	0.000	22.53733	36.49231
Number of children < 5 years			−.0010666	.000149	−7.16	0.000	−.0013587	−.0007746
District								
Baghpat (Index)			−	−	−	−	−	−
Bareilly			−9.061472	4.103751	−2.21	0.027	−17.10468	−1.018268
Mau			2.170310	4.120645	0.53	0.598	−5.906006	10.24663
Meerut			−11.33141	4.039701	−2.81	0.005	−19.24908	−3.413747
Moradabad			−.9637972	3.560949	−0.27	0.787	−7.943130	6.015536
Muzafarnagar			3.919320	3.749419	1.05	0.296	−3.429405	11.26805
Rampur			−10.62098	4.348856	−2.44	0.015	−19.14458	−2.097374
Saharanpur			−4.534669	4.301110	−1.05	0.292	−12.96469	3.895352
Shahjahanpur			8.714352	4.190468	2.08	0.038	.5011852	16.92752
Sitapur			17.53107	4.069808	4.31	0.000	9.554398	25.50775
Number of Mosque Announcements (Quartile, Percentile)								
1	< 25th	(Index)	−	−	−	−	−	−
2	25–50th		1.831605	1.056808	1.73	0.083	-.2396992	3.90291
3	50–75th		2.450691	1.428741	1.72	0.086	-.3495898	5.250972
4	> 75th		4.822117	1.515839	3.18	0.001	1.851128	7.793106
Number of Rallies (Quartile, Percentile)								
1	< 25th	(Index)	−	−	−	−	−	−
2	25–50th		−1.591375	.7870536	−2.02	0.043	−3.133972	−.0487782
3	50–75th		−3.412405	.8425505	−4.05	0.000	−5.063774	−1.761037
4	> 75th		−2.612224	.9626774	−2.71	0.007	−4.499037	−.7254114
Number of *Bullawa Tollies* (Quartile, Percentile)								
1	< 25th	(Index)	−	−	−	−	−	−
2	25–50th		.3147572	.968306	0.33	0.745	−1.583088	2.212602
3	50-75th		2.853786	1.106337	2.58	0.010	.6854046	5.022167
4	> 75th		4.161977	1.26913	3.28	0.001	1.674527	6.649426
Variance of fixed effects:			33.933216	1.6500424				
Variance of random effects			41.29389	9.0494798				

* Coefficients and standard errors adjusted for differences between Blocks and changes within Block values over time (time-varying covariate values at the Block level) by using a Generalized Linear Latent And Mixed Model (GLLAMM).

Predictions about the difference in booth coverage between CMC and Non-CMC areas (adjusted based on the model estimates in Table 4) are shown in Table 5. The predictions are done separately for each district and assume that the number of children under five is the average number across the blocks in the district across the study period. Although the number of rallies was a significant determinant of the outcome in this analysis, it was dropped from the prediction because the relationship was negative. For these predictions, we compare our outcome measure in four different situations: (1) the number of social mobilization activities (mosque announcements, *Bullawa Tollies*) in the CMC areas of a block is within the range of the first quartile (less than the 25th percentile); (2) the number of mosque announcements is within the range of the 4th quartile (75th percentile or higher) and the number of *Bullawa Tollies* within the range of the 1st quartile; (3) the number of mosque announcements is within the range of the 1st quartile and the number of *Bullawa Tollies* within the range of the 4th quartile; and, (4) both the number of mosque announcements and the number of *Bullawa Tollies* are within the range of the 4th quartile. The results show that the outcome measure (difference in booth coverage between CMC and Non-CMC areas) varies considerably between districts (a range of about 30 percentage points between highest and lowest). More importantly, the results also show that when the number of mosque announcements and *Bullawa Tollies* in a block is within the 4th quartile range (greater than 48 and 43, respectively) the difference in booth coverage between CMC and Non-CMC areas increases nine percentage points compared to when the number of these activities is within the 1st quartile range.

**Table 5:** Predicted difference in booth coverage (%) between CMC and non-CMC areas of a block during supplemental immunization activities in Uttar Pradesh, India by number of social mobilization activities carried out and district*

**District**		**Number of Social Mobilization Activities per SIA**			
		**Index (< 31 Mosque Announcements + < 33 Bullawa Tollies)**	**> 48 Mosque Announcements vs. Index**	**> 43 Bullawa Tollies vs. Index**	**> 48 Mosque Announcements + > 43 Bullawa Tollies vs. Index**
1	Baghpat	17.0	21.8	21.1	25.9
2	Bareilly	10.6	15.4	14.8	19.6
3	Mau	21.0	25.9	25.2	30.0
4	Meerut	8.5	13.3	12.6	17.5
5	Moradabad	10.4	15.2	14.5	19.3
6	Muzafarnagar	19.1	23.9	23.2	28.0
7	Rampur	8.3	13.2	12.5	17.4
8	Saharanpur	14.8	19.7	19.0	23.8
9	Shahjahanpur	26.0	30.8	30.2	35.0
10	Sitapur	37.3	42.1	41.5	46.3

* Predictions are based on post-estimation linear combinations of estimates in model in Table 4 above. These predictions are adjusted for the District in which a Block is located, the average number of children less than five years of age across Blocks in a District, and the differences between Blocks and changes within Block values over time (time-varying covariate values at the Block level for number of social mobilization activities) by using a Generalized Linear Latent And Mixed Model (GLLAMM).

### Determinants of X to P conversion

Determinants of the difference in conversion of X houses to P between CMC and Non-CMC areas are shown in Table 6. Covariates originally entered into the model are the same as above. The number of mosque announcements was the only significant social mobilization determinant of the difference in conversion of X house to P between CMC and Non-CMC areas. The district was also a significant determinant. *Bullawa Tollies* appears to be an important social mobilization determinant of this outcome but it was not significant at the .05 level (p = .061). The number of children under five years of age also appears to be an important determinant but not significant at the .05 level either (p = .063). Because the p-values of *Bullawa Tollies* and the number of children less than five were less than 0.1, these items were kept in the model.

**Table 6:** Determinants of the difference in percent of X houses converted to P between CMC and non-CMC areas of a block*

**Variable**			**Coefficient**	**Std. Err.**	**z**	**p value**	**[95% Confidence Interval]**	
(Constant)			12.14412	4.70592	2.58	0.010	2.920682	21.36755
Number of children < 5 years			−.0006623	.0003569	−1.86	0.063	−.0013618	.0000372
District								
1	Baghpat (Index)		−	−	−	−	−	−
2	Bareilly		−2.262005	1.151339	−1.96	0.049	−4.518589	−.0054215
3	Mau		6.324774	1.667669	3.79	0.000	3.056204	9.593345
4	Meerut		−10.04491	.8974011	−11.19	0.000	−11.80379	−8.286038
5	Moradabad		−4.138752	1.738614	−2.38	0.017	−7.546373	−.7311312
6	Muzafarnagar		−5.157229	1.138899	−4.53	0.000	−7.389431	−2.925028
7	Rampur		−7.979791	.4193138	−19.03	0.000	−8.801630	−7.157951
8	Saharanpur		−9.242826	1.04674	−8.83	0.000	−11.29440	−7.191252
9	Shahjahanpur		2.645487	2.01286	1.31	0.189	−1.299647	6.590620
	Sitapur		−1.617467	3.03375	−0.53	0.594	−7.563508	4.328574
Number of Mosque Announcements (Quartile, Percentile)								
1	< 25th	(Index)	−	−	−	−	−	−
2	25–50th		3.280323	1.684365	1.95	0.051	−.020971	6.581618
3	50-75th		4.814767	1.924814	2.50	0.012	1.042201	8.587334
4	> 75th		4.742434	2.12667	2.23	0.026	.5742373	8.910632
Number of *Bullawa Tollies* (Quartile, Percentile)								
1	< 25th	(Index)	−	−	−	−	−	−
2	25–50th		.1520856	.8256602	0.18	0.854	−1.466179	1.770350
3	50-75th		1.929366	1.24142	1.55	0.120	−.5037729	4.362504
4	> 75th		3.365150	1.794372	1.88	0.061	−.1517546	6.882054
Variance of fixed effects:			43.142147	6.7934335				
Variance of random effects			14.77656	5.8608284				

* Coefficients and standard errors adjusted for differences between Blocks and changes within Block values over time (time-varying covariate values at the Block level) by using a Generalized Linear Latent And Mixed Model (GLLAMM).

Table 7 displays the adjusted predictions about the difference in conversion of X houses to P between CMC and Non-CMC areas (based on the model estimates in Table 6). The predictions are carried out as above. The results show that the outcome measure (difference in conversion of X houses to P between CMC and Non-CMC areas) also varies by district with a range of about 15 percentage points between highest and lowest. Most important, when the number of mosque announcements and *Bullawa Tollies* in the CMC areas of a block is within the 4th quartile range (greater than 48 and 43, respectively) the difference in X to P conversion between CMC and Non-CMC areas of a block is about eight percentage points compared to when the number of these activities is within the 1st quartile range.

**Table 7:** Predicted difference in percent of X houses converted to P (%) between CMC and non-CMC areas of a block during Supplemental immunization activities in Uttar Pradesh, India by number of social mobilization activities carried out and district*

**District**		**Number of Social Mobilization Activities per SIA**			
		**Index (< 31 Mosque Announcements + < 33 Bullawa Tollies)**	**> 48 Mosque Announcements vs. Index**	**> 43 Bullawa Tollies vs. Index**	**> 48 Mosque Announcements + > 43 Bullawa Tollies vs. Index**
1	Baghpat	4.3	9.1	7.7	12.5
2	Bareilly	3.8	8.5	7.1	11.9
3	Mau	11.9	16.6	15.2	20.0
4	Meerut	−3.9	0.8	−0.6	4.2
5	Moradabad	−3.3	1.5	0.1	4.8
6	Muzafarnagar	−1.9	2.8	1.4	6.2
7	Rampur	−2.4	2.4	1.0	5.7
8	Saharanpur	−3.4	1.3	0.0	4.7
9	Shahjahanpur	7.2	11.9	10.6	15.3
10	Sitapur	4.5	9.2	7.8	12.6

* Predictions are based on post-estimation linear combinations of estimates in model in Table 6 above. These predictions are adjusted for the District in which a Block is located, the average number of children less than five years of age across Blocks in a District, and the differences between Blocks and changes within Block values over time (time-varying covariate values at the Block level for number of social mobilization activities) by using a Generalized Linear Latent And Mixed Model (GLLAMM).

## Discussion

### Limitations

The data and analysis have several limitations. First, there is a limitation in assessing the effects of social mobilization on performance of supplementary immunization activities such as national or sub-national immunization days. SIAs, while necessary, are not sufficient. Many other factors affect progress of the polio eradication effort such as routine immunization efforts, sanitation, and vaccine efficacy in crowded, unsanitary areas.

Second, the SM Net and CGPP carry out more social mobilization activities than documented here. For example, community mobilization coordinators (CMCs) arrange for influential people (influencers) to visit the homes of families who are resistant to vaccination for the purpose of encouraging vaccination of the families’ children. These homes are classified as X houses in the analysis above. The number of influential persons who visited homes, or the number of resistant homes (X houses) visited by influencers was not documented and could therefore not be included in the analysis.

Third, the social mobilization determinants in our analysis were the counts of activities. We do not include information about the quality of these activities. It is likely that quality is as much or more important than the quantity of activities and that for some activities an increase in number may lead to lower quality and effectiveness. Quality measures of social mobilization activities should be investigated in the future as to how the determine vaccination performance.

### Determinants of the difference in booth coverage between CMC and Non-CMC areas

The district in which an SIA is carried out affects the predicted outcome. Further investigation and analysis to determine the factors associated with this variation by district would be useful for findings ways to improve booth coverage and conversion of X houses to P in lower performing districts. The CGPP and SM Net should conduct sufficient numbers of mosque announcements and *Bullawa Tollies* in a block in preparation for each SIA (within the range of the 4th quartile presented here). There is evidence that a higher number of rallies is actually detrimental to the objective of increasing booth coverage. We cannot identify a clear reason for this finding about rallies and recommend placing more emphasis on fewer high quality rallies.

The other social mobilization activities analyzed here (influencer meeting, IPC meetings, temple announcements) do not appear to have direct effects on booth coverage or conversion of X houses to P. These activities may be useful for other reasons (e.g., IPC meetings may improve other outcomes such as routine immunization coverage). Or, these activities may have indirect effects on the outcomes studied here. For example, influencer meetings may lead to more visits by influential people to resistant households which may have a direct effect on conversion of X houses to P. However, the number of X houses visited by influential people was not available for this analysis.

### Policy implications

The SM Net and CGPP should ensure appropriate numbers of mosque announcements, *Bullawa Tollies* and rallies are carried out in preparation of each SIA. The recommendation is that more than 48 mosque announcements and 43 *Bullawa Tollies* be carried out in the CMCs areas of each block for each SIA. The time period is usually within the month prior to an SIA. Each block should carry out no more than 16 rallies and should consider focusing more on the quality than the quantity of the rallies, or consider shifting efforts away from rallies altogether to mosque announcements and *Bullawa Tollies*. Documenting social mobilization activities that are not now being documented would allow evaluation of these other activities.

## Conclusions

Social mobilization activities can improve the performance of mass vaccination campaigns. In CGPP districts, mosque announcements and *Bullawa Tollies* were important determinants of desired SIA outcomes. The CGPP and SM Net should conduct sufficient numbers of mosque announcements and *Bullawa Tollies* in each block in preparation for each SIA (within the range of the 4th quartile presented here). High numbers of social mobilization activities are not always beneficial, however. There is evidence that a high number of rallies wereactually detrimental to the objective of increasing booth coverage in the study setting. Study of the effects of social mobilization activities is important for helping rationalize how scarce resources are used in improving health and development in low and middle income countries. Quality measures of social mobilization activities should be investigated in the future as to how they determine OPV campaign performance.

## Endnotes

^a^The CORE Group works in the following 10 districts of Uttar Pradesh, India: Baghpat, Bareilly, Meerut, Muzaffarnagar, Moradabad, Mau, Rampur, Saharanpur, Shahjahanpur and Sitapur (total of 56 blocks).

## Competing interests

All authors have received salary support from the US Agency for International Development (USAID) under Cooperative Agreement GHN-A-00-07-00014. This salary support has covered implementation of the project described and/or for writing this manuscript.

## Authors’ contributions

WW wrote key sections of the Methods, Results, Discussions and Conclusions. He also designed and carried out exploratory and statistical analysis. MHR wrote key sections of the Methods and assisted with analysis of longitudinal data. RS wrote key sections of the Background and edited the manuscript. DW edited the manuscript and assisted in the design of the analysis. All authors have read and approved the final version of the manuscript.

## Pre-publication history

The pre-publication history for this paper can be accessed here:

http://www.biomedcentral.com/1471-2334/13/17/prepub
